# Plasma cell free next-generation sequencing detects an unusual pneumonia pathogen in an immunocompetent adolescent with acute respiratory distress syndrome

**DOI:** 10.3389/fped.2022.1034632

**Published:** 2022-12-05

**Authors:** Katherine M. Rodriguez, Nanda Ramchandar, Nicole G. Coufal

**Affiliations:** ^1^ Division of Pediatric Critical Care, Department of Pediatrics, University of California, San Diego, CA, United States; ^2^ Division of Pediatric Critical Care, Department of Pediatrics, Rady Children's Hospital San Diego, San Diego, CA, United States; ^3^ Division of Infectious Disease, Department of Pediatrics, Navy Medical Center, San Diego, CA, United States; ^4^ Division of Infectious Disease, Department of Pediatrics San Diego, Rady Children's Hospital San Diego, San Diego, CA, United States

**Keywords:** plasma microbial cell-free DNA next generation sequencing, pneumonia, acute respiratory distress syndrome, pediatric critical care, legionella

## Abstract

This case details a rapid diagnosis of legionella pneumonia causing severe acute respiratory distress syndrome (ARDS) in an otherwise healthy adolescent through plasma microbial cell-free DNA next generation sequencing (mcfDNA-NGS). Diagnosis by mcfDNA-NGS of this unexpected pathogen led to narrowing of antimicrobials and the addition of glucocorticoids as adjunctive therapy for ARDS.

## Introduction

Community acquired pneumonia (CAP) is a leading cause of hospitalization in children. Pediatric pneumonia is predominantly attributable to viral causes such as influenza and respiratory syncytial virus. Bacterial causes of pneumonia are primarily due to *Streptococcus pneumonia*, group A Streptococcus, or *Staphylococcus aureus*. The most common atypical cause of pneumonia is *Mycoplasma pneumonia* (1). Isolating bacterial pathogens in cases of pneumonia is historically challenging with only 17%–21% of blood cultures identifying a bacterial pathogen (2, 3). Sensitivity of blood culture is greatly affected by antibiotic pretreatment. Cultures from pleural fluid tend to have a higher diagnostic yield but invasive procedures, such as thoracentesis and bronchoalveolar lavage, are less commonly performed in pediatrics. This case details the detection of an unusual pathogen *via* plasma microbial cell-free DNA next generation sequencing (mcfDNA-NGS) in an immunocompetent patient with severe acute respiratory distress syndrome (ARDS).

## Patient presentation

**Figure 1:**
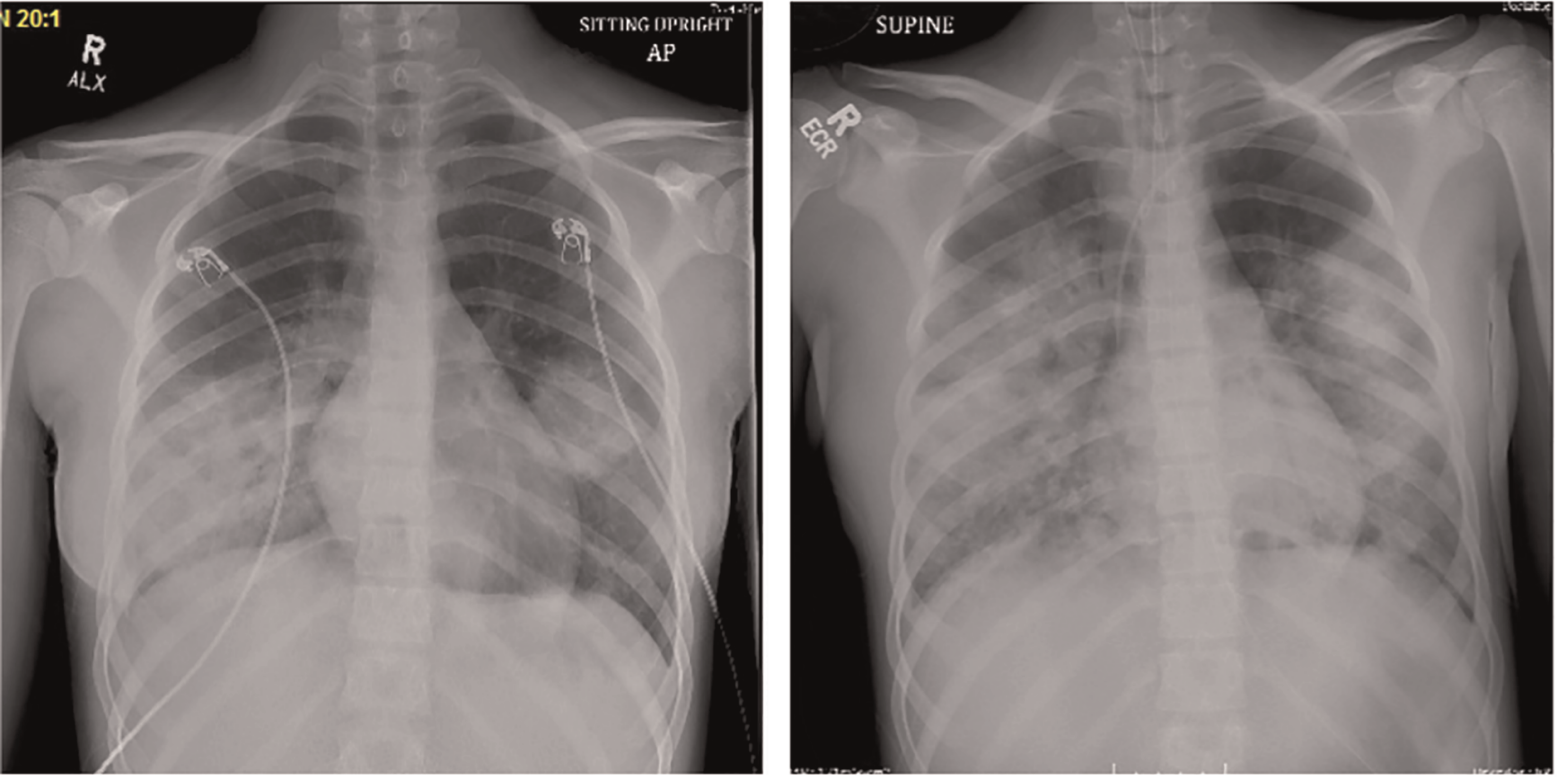
Chest radiograph on HD 1 (left). Chest radiograph on HD 4, post-intubation (right).

A previously healthy 14-year-old female presented to the Emergency Department with cough, shortness of breath, and fever to 38.8°C. The patient reported 12 days of cough and rhinorrhea with symptomatic worsening five days prior to presentation, including: fatigue, nausea, vomiting, diarrhea, abdominal pain, and headache. The patient was fully vaccinated (including against SARS-CoV-2), did not have known exposure to sick contacts, and denied any recent travel from her home in Southern California. She had no underlying immunodeficiency nor was she receiving immunosuppressive therapy. She denied exposure to unpasteurized dairy, water parks, freshwater swimming, or ocean swimming. She endorsed occasional remote hot tub use, with no use in the 2 weeks prior to presentation. The patient was admitted and initiated on supplemental high flow oxygen due to hypoxia and increased work of breathing. Initial laboratory studies were significant for a C-reactive protein of 31.4 mg/dl (ref 0–0.99 mg/dl), a procalcitonin of 29.57 ng/ml (ref <0.5 ng/ml), leukocytosis with neutrophilic predominance (WBC 17.3, 90% neutrophils, 7% bands), and a respiratory multiplex PCR panel (ePLEX, Genmark Diagnostics, Carlsbad, CA) from the nasopharynx positive for human rhinovirus/enterovirus. A chest radiograph (CXR) on admission was notable for bilateral opacities consistent with multifocal pneumonia (Figure 1). She was initiated on ceftriaxone for empiric coverage of CAP. On hospital day (HD) 2, due to persistent high fever (40°C) and worsening respiratory status, clindamycin was added for coverage of methicillin resistant staph aureus (MRSA) and anaerobic pathogens. Local MRSA isolates demonstrate 84% sensitivity to clindamycin.

On HD 3, the patient exhibited worsening respiratory failure necessitating transfer to the Pediatric Intensive Care Unit and bilevel positive airway pressure (BIPAP). She had worsening pleural effusions on CXR and clinical signs of fluid overload thus what started on intermittent intravenous furosemide. Ultimately, her hypoxia and work of breathing necessitated intubation on HD4. At that time, she met clinical criteria for severe ARDS with an initial oxygenation index (OI) of 30 and a maximum OI of 39, a PaO2/FiO2 ratio of 90, with a maximum positive end-expiratory pressure (PEEP) of 24. Adjuncts such as inhaled nitric oxide and prone positioning were required for refractory hypoxemia. Bronchoalveolar lavage was not performed for diagnostic purposes due hypoxia and the severity of her ARDS. She was hemodynamically stable during this period with intermittent use of low dose epinephrine infusion to support blood pressure and diuresis. On HD 3 levofloxacin was added to the antimicrobial regimen of ceftriaxone and clindamycin to cover for atypical organisms.

On HD 3 plasma mcfDNA-NGS (Karius Inc., Redwood City, CA) was sent, this is common practice at our institution for critically ill patients where infectious cause is suspected but the pathogen has not been identified by standard testing. Plasma mcfDNA-NGS resulted positive after 46 h on HD 5 for *Legionella pneumophilia*. The organism met the sequencing coverage threshold for reporting, but not for quantification and thus molecules per microliter was not given. Once the pathogen was identified, antimicrobials were narrowed to levofloxacin alone, the treatment of choice for severe legionella infection (4). The patient was also initiated on high dose glucocorticoids as there is some evidence of benefit in severe ARDS secondary to legionella (4). Pathogen identification was later confirmed *via* an atypical pneumonia PCR panel (Quest Diagnostics, San Juan Capistrano, CA) sent from an endotracheal tube aspirate after intubation and a urine legionella antigen test (Quest Diagnostics, San Juan Capistrano, CA). These tests were sent on HD 4 and resulted after 66 and 73 h respectively on HD7 after mcfDNA-NGS results were known. Legionella was not detected *via* standard blood culture or bacterial respiratory smear/culture.

Given the unusual Legionella diagnosis, an immunology consultation was obtained, identifying a slightly low immunoglobulin G of 647 mg/dl (ref 749–1,640 mg/dl) and lymphopenia with absolute lymphocyte count of 957 cells/ul (ref 1200–5200 cells/ul), both determined to be most likely attributable to the acute infection. An HIV antigen/antibody 4th generation test was non-reactive, and immunoglobulin A and immunoglobulin M antibody levels were normal. After five days of mechanical ventilation the patient was successfully extubated. She was treated for a total 14-day course of levofloxacin and was weaned off all respiratory support prior to discharge. An investigation by local public health authorities failed to identify a source for her legionella infection.

## Discussion

Pneumonia is common in the pediatric population, though rarely caused by Legionella. In the pediatric population legionella pneumonia can occur in infants or patients with immunosuppression and is often nosocomially acquired (5). The patient described had a severe and unusual course for a healthy child without any specific exposures, although hot tub usage is a known risk in individuals over age 45 or heavy smokers (6). Additionally, this case was reported to the county health department and an associated cluster of legionella infection was not identified. Empiric parenteral therapy commonly prescribed for CAP such as ampicillin or ceftriaxone are inactive against this intracellular pathogen. Macrolides, such as azithromycin, can be effective in the treatment of mild to moderate disease. Fluoroquinolones such as levofloxacin provide the best intrinsic activity against Legionella and are recommended in severe illness (4). The low sensitivity of blood cultures and standard respiratory cultures for detecting Legionella may result in missed diagnoses, and in serious cases, may result in increased morbidity due to inadequate empiric therapy or overly broad empiric therapy.

Traditional methods to diagnose Legionella include lower respiratory tract culture, urinary antigen testing, and quantitative PCR testing of serum, urine, or respiratory samples. Lower respiratory tract culture is considered the gold standard but has a limited and variable sensitivity of 10%–80% (4, 7) and can take up to 7 days to result. Urinary antigen detection has a sensitivity of 56%–99% (7) but is most useful in detecting the Lp1 subtype and has decreased sensitivity with other subtypes. PCR sensitivity ranges from 33%–70% depending on sample source and is highest in lower respiratory tract samples. All methods have high specificity of 98%–100% (4). There are 3 cases of legionella diagnosed by metagenomic NGS (mNGS) described in the literature including one immunosuppressed child (8) and two adults (9, 10). In the case described by Wang et al. (8) evidence of legionella infection was also found in the bronchial alveolar lavage (BAL) fluid which was positive by gram stain, mNGS, and culture. Legionella was confirmed by Yi et al. (9) with positive mNGS and PCR testing from sputum and pleural fluid. Yue et al. (10) also detected legionella by mNGS of BAL fluid and later detected anti-legionella IgM antibodies. Blood cultures were negative in all three cases.

In case series where plasma mcfDNA-NGS has been applied in complicated CAP, the addition of mcfDNA-NGS to standard of care diagnostics improved bacterial diagnosis from 27% by conventional culture methods to 86% by mcfDNA-NGS (11). Identifying a causative organism allows antibiotic selection to be narrowed limiting the patient exposure to unnecessarily broad antibiotics. Importantly, the rapid pathogen identification allowed the clinical team to make decisions about initiation of adjunctive treatment with steroids, which are currently not routinely recommended in the pediatric ARDS treatment guidelines (12). The turn-around time for the mcfDNA-NGS test was less than 48-h and allowed the clinical team to rapidly optimize therapy.

Legionella infection, while not uncommon in adults, is quite rare in children. While currently not the standard of care, the application of plasma mcfDNA-NGS in critically ill children with pneumonia can aid in pathogen identification, especially where disease severity or clinical complications precludes other well established but invasive diagnostic modalities. It was through the quick and effective application of plasma mcfDNA-NGS that this pathogen was detected. Early detection allowed for narrowing of antibiotics and facilitated management of severe ARDS leading to a favorable outcome.

## Data Availability

The original contributions presented in the study are included in the article/Supplementary Material, further inquiries can be directed to the corresponding author/s.

## References

[1] Bradley JS Byington C Shah S Alverson B Carter E Harrison C The management of community-acquired pneumonia in infants and children older than 3 months of age: clinical practice guidelines by the pediatric infectious diseases society and the infectious diseases society of America. Clin Infect Dis. (2011) 53(7):e25–76. 10.1093/cid/cir531 21880587 PMC7107838

[2] Stankey CT Spaulding AB Doucette A Hamre KES Wheeler W Pomputius WF Blood culture and pleural fluid culture yields in pediatric empyema patients. Pediatr Infect Dis J. (2018) 37(9):952–4. 10.1097/INF.0000000000001940 29438130

[3] Erlichman I Breuer O Shoseyov D Cohen-Cymberknoh M Koplewitz B Averbuch D Complicated community acquired pneumonia in childhood: different types, clinical course, and outcome. Pediatr Pulmonol. (2017) 52(2):247–54. 10.1002/ppul.23523 27392317

[4] Roig J. Legionnaires’ disease: a rational approach to therapy. J Antimicrob Chemother. (2003) 51(5):1119–29. 10.1093/jac/dkg191 12668578

[5] Fulova M Kotrbancova M Brazinova A Boledovicova J Trnkova K Spalekova M. Legionnaires’ disease in pediatric patients, control measures and 5-year follow-up. Pediatr Infect Dis J. (2020) 39(11):990–4. 10.1097/INF.0000000000002781 32472821

[6] Ahmed M Mustfa N. Hot tub legionella pneumonia outbreak. Eur Respir J. (2014) 44(5):1379–81. 10.1183/09031936.00005114 25362129

[7] Cunha BA Burillo A Bouza E. Legionnaires’ disease. Lancet. (2016) 387(10016):376–85. 10.1016/S0140-6736(15)60078-2 26231463

[8] Wang Y Dai Y Lu H Chang W Ma F Wang Z Case report: metagenomic next-generation sequencing in diagnosis of legionella pneumophila pneumonia in a patient after umbilical cord blood stem cell transplantation. Front Med. (2021) 8:643473. 10.3389/fmed.2021.643473 PMC8232522 34179036

[9] Yi H Fang J Huang J Liu B Qu J Zhou M. Legionella pneumophila as cause of severe community-acquired pneumonia, China. Emerg Infect Dis. (2020) 26(1):160–2. 10.3201/eid2601.190655 31855541 PMC6924908

[10] Yue R Wu X Li T Chang L Huang X Pan L Early detection of legionella pneumophila and aspergillus by mNGS in a critically ill patient with Legionella pneumonia after extracorporeal membrane oxygenation treatment: case report and literature review. Front Med. (2021) 8:686512. 10.3389/fmed.2021.686512 PMC8277993 34277662

[11] Farnaes L Wilke J Ryan Loker K Bradley JS Cannavino CR Hong DK Community-acquired pneumonia in children: cell-free plasma sequencing for diagnosis and management. Diagn Microbiol Infect Dis. (2019) 94(2):188–91. 10.1016/j.diagmicrobio.2018.12.016 30819624 PMC7125591

[12] Orloff KE Turner DA Rehder KJ. The current state of pediatric acute respiratory distress syndrome. Pediatr Allergy Immunol Pulmonol. (2019) 32(2):35–44. 10.1089/ped.2019.0999 31236307 PMC6589490

